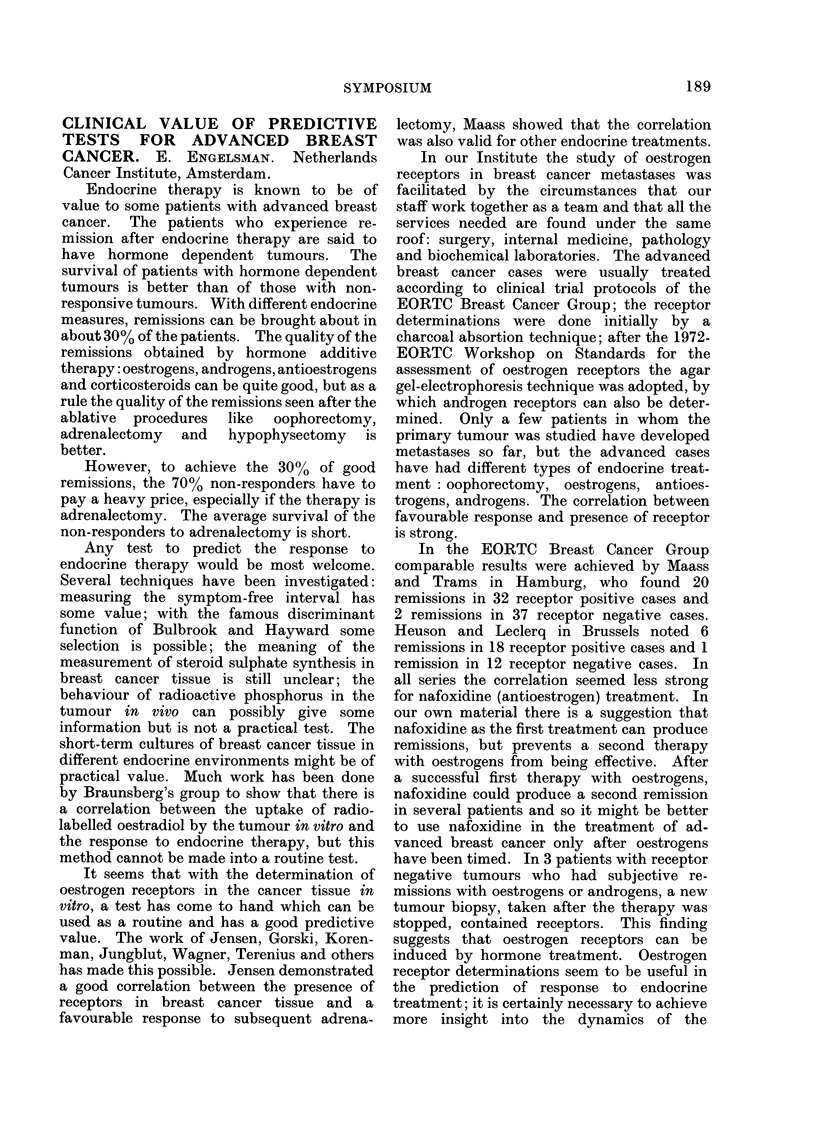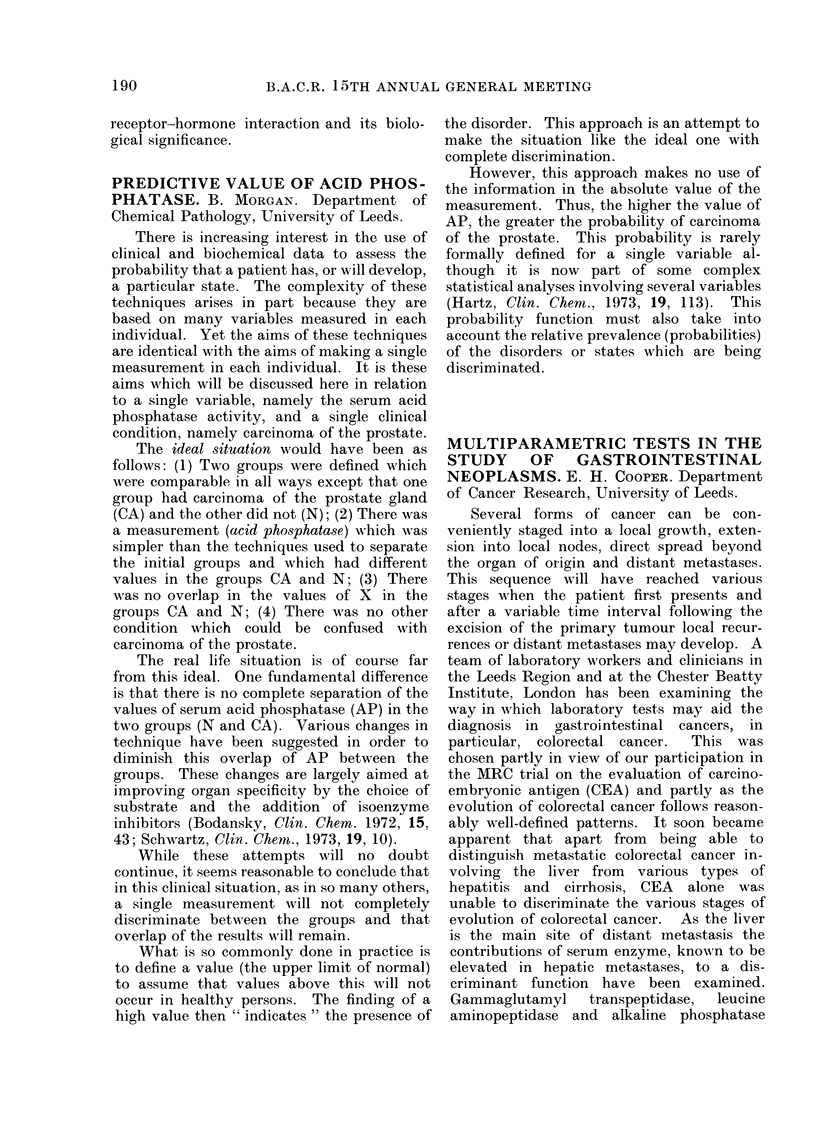# Proceedings: Clinical value of predictive tests for advanced breast cancer.

**DOI:** 10.1038/bjc.1974.178

**Published:** 1974-08

**Authors:** E. Engelsman


**CLINICAL VALUE OF PREDICTIVE TESTS FOR ADVANCED BREAST CANCER.** E. ENGELSMAN. Netherlands Cancer Institute, Amsterdam.

Endocrine therapy is known to be of value to some patients with advanced breast cancer. The patients who experience remission after endocrine therapy are said to have hormone dependent tumours. The survival of patients with hormone dependent tumours is better than of those with non-responsive tumours. With different endocrine measures, remissions can be brought about in about 30% of the patients. The quality of the remissions obtained by hormone additive therapy: oestrogens, androgens, antioestrogens and corticosteroids can be quite good, but as a rule the quality of the remissions seen after the ablative procedures like oophorectomy, adrenalectomy and hypophysectomy is better.

However, to achieve the 30% of good remissions, the 70% non-responders have to pay a heavy price, especially if the therapy is adrenalectomy. The average survival of the non-responders to adrenalectomy is short.

Any test to predict the response to endocrine therapy would be most welcome. Several techniques have been investigated: measuring the symptom-free interval has some value; with the famous discriminant function of Bulbrook and Hayward some selection is possible; the meaning of the measurement of steroid sulphate synthesis in breast cancer tissue is still unclear; the behaviour of radioactive phosphorus in the tumour *in vivo* can possibly give some information but is not a practical test. The short-term cultures of breast cancer tissue in different endocrine environments might be of practical value. Much work has been done by Braunsberg's group to show that there is a correlation between the uptake of radio-labelled oestradiol by the tumour *in vitro* and the response to endocrine therapy, but this method cannot be made into a routine test.

It seems that with the determination of oestrogen receptors in the cancer tissue *in vitro*, a test has come to hand which can be used as a routine and has a good predictive value. The work of Jensen, Gorski, Korenman, Jungblut, Wagner, Terenius and others has made this possible. Jensen demonstrated a good correlation between the presence of receptors in breast cancer tissue and a favourable response to subsequent adrenalectomy, Maass showed that the correlation was also valid for other endocrine treatments.

In our Institute the study of oestrogen receptors in breast cancer metastases was facilitated by the circumstances that our staff work together as a team and that all the services needed are found under the same roof: surgery, internal medicine, pathology and biochemical laboratories. The advanced breast cancer cases were usually treated according to clinical trial protocols of the EORTC Breast Cancer Group; the receptor determinations were done initially by a charcoal absorption technique; after the 1972-EORTC Workshop on Standards for the assessment of oestrogen receptors the agar gel-electrophoresis technique was adopted, by which androgen receptors can also be determined. Only a few patients in whom the primary tumour was studied have developed metastases so far, but the advanced cases have had different types of endocrine treatment : oophorectomy, oestrogens, antioestrogens, androgens. The correlation between favourable response and presence of receptor is strong.

In the EORTC Breast Cancer Group comparable results were achieved by Maass and Trams in Hamburg, who found 20 remissions in 32 receptor positive cases and 2 remissions in 37 receptor negative cases. Heuson and Leclerq in Brussels noted 6 remissions in 18 receptor positive cases and 1 remission in 12 receptor negative cases. In all series the correlation seemed less strong for nafoxidine (antioestrogen) treatment. In our own material there is a suggestion that nafoxidine as the first treatment can produce remissions, but prevents a second therapy with oestrogens from being effective. After a successful first therapy with oestrogens, nafoxidine could produce a second remission in several patients and so it might be better to use nafoxidine in the treatment of advanced breast cancer only after oestrogens have been timed. In 3 patients with receptor negative tumours who had subjective remissions with oestrogens or androgens, a new tumour biopsy, taken after the therapy was stopped, contained receptors. This finding suggests that oestrogen receptors can be induced by hormone treatment. Oestrogen receptor determinations seem to be useful in the prediction of response to endocrine treatment; it is certainly necessary to achieve more insight into the dynamics of the receptor–hormone interaction and its biological significance.